# A Scoping Review and Prevalence Analysis of Soil-Transmitted Helminth Infections in Honduras

**DOI:** 10.1371/journal.pntd.0002653

**Published:** 2014-01-23

**Authors:** Ana Lourdes Sanchez, José Antonio Gabrie, María Mercedes Rueda, Rosa Elena Mejia, Maria Elena Bottazzi, Maritza Canales

**Affiliations:** 1 Department of Health Sciences, Brock University, St. Catharines, Ontario, Canada; 2 School of Microbiology, National Autonomous University of Honduras (UNAH), Tegucigalpa, Honduras; 3 National Surveillance Laboratory, Honduras Ministry of Health, Tegucigalpa, Honduras; 4 National School of Tropical Medicine, Baylor College of Medicine, Houston, Texas, United States of America; Swiss Tropical and Public Health Institute, Switzerland

## Abstract

**Background:**

Honduras is endemic for soil-transmitted helminth (STH) infections, but critical information gaps still remain on the prevalence and intensity of these infections as well as on their spatial distribution at subnational levels.

**Objectives:**

Firstly, to review the research activity on STH infections in Honduras and secondly, to carry out a national prevalence analysis and map the geographical distribution of these infections in children.

**Methods:**

A systematic search was conducted of the published and grey literature to identify scientific work on the impact and prevalence of STH infections done between May 1930 and June 30, 2012. International databases and Honduran journals were searched. Grey literature was gleaned from local libraries and key informants. Select studies conducted between 2001 and 2012 were used to produce prevalence maps and to investigate association between STH prevalence and socio-economic and environmental factors.

**Results:**

Of 257 identified studies, 211 (21.4% peer-reviewed) were retained for analysis and categorized as clinical research (10.9%), treatment efficacy studies (8.1%) or epidemiological studies (81%). Prevalence analysis and geographical mapping included 36 epidemiological studies from Honduras's 18 departments and 23% of its municipalities. Overall STH prevalence was >50% in 40.6% of municipalities. Prevalences above 20% for each trichuriasis, ascariasis, and hookworm infection were found in 68%, 47.8%, and 7.2% of studied municipalities, respectively. Municipalities with lower human development index, less access to of potable water, and with higher annual precipitation showed higher STH prevalences.

**Conclusions:**

This is the first study to provide a comprehensive historic review of STH research activity and prevalence in Honduras, revealing important knowledge gaps related to infection risk factors, disease burden, and anti-parasitic drug efficacy, among others. Our decade-long prevalence analysis reveals geographical differences in STH prevalence and these findings suggest that differential intervention strategies might be necessary in Honduras for the control of these infections.

## Author Summary

Soil-transmitted helminthiases are infections of public health importance in Honduras. Although research data on this topic in the country is somewhat inconsistent and scattered, a wealth of information has been produced by Honduran researchers and practitioners over the years. The vast majority of this information, however, remains inaccessible in the gray literature, thus missing its potential to inform research, policy, and practice. We undertook a thorough review of the literature produced from 1930 to 2012 in order to identify research gaps and, based on a select number of studies, conducted a prevalence analysis with data generated between 2001 and 2012. Our study reveals that these parasitic infections have not been a focus of sustained research efforts in Honduras. Literature analysis identified current knowledge gaps related to infection risk factors, disease burden, and anti-parasitic drug efficacy, among others. Evaluating and monitoring the national control program also emerged as a priority. Our study also reveals geographic areas of very high endemicity, suggesting that targeted interventions might be appropriate in localized areas of the country. Honduras is at present expanding the deworming program to include pre-school children and the data presented here could serve as a baseline for future monitoring and research.

## Introduction

Intestinal parasites including soil-transmitted helminths (STH) have been long recognized as public health problems in much of the developing world [1]. Soil-transmitted helminthiases are caused by four species of intestinal worms, namely, *Ascaris lumbricoides*, *Trichuris trichiura*, and the two hookworm species *Ancylostoma duodenale* and *Necator americanus* [2]. Current estimates show that more than 2 billion people worldwide are infected with STH, the majority residing in low and middle income countries (LMICs) [3], [4]. Within LMICs, STH endemicity is concentrated in and around areas where ecological and environmental characteristics intersect with conditions that facilitate transmission [5], [6]. In addition, recent studies have shown that soil-transmitted helminthiases and other neglected tropical diseases (NTDs) also cause a great burden among poor populations living in wealthy nations, including the United States [7], [8]. Primarily and due to their widespread distribution and chronic nature, STH infections pose a high burden in endemic populations [2], [4], [9] among which, children and youth are disproportionally affected [10], [11]. Hence, global efforts are currently focused on this vulnerable population [4]. As a highly effective and immediate intervention for reducing STH morbidity in high risk groups, the World Health organization (WHO) recommends to all disease-endemic countries periodic administration of anthelminthic medication (a strategy called preventive chemotherapy, PC) [12], [13]. Implementing PC is, however, not without challenges. Barry et al (2013) recently calculated that for 2011, 875 million children (70% of school age) lived in high-risk areas worldwide but only 38% of pre-school and 34% of school age children were reached by PC activities [14]. Aware of this gap, and in support of WHO's roadmap to guide implementation of NTD's control strategies [15], a number of private and public partners have agreed to join forces to assure that anthelminthic drugs and other interventions reach all people suffering from NTDs including STH infections [16].

Honduras, as most countries in Latin America, is endemic for STH infections [4], [5], [17], [18]. Organized efforts to control these infections in the country can be traced back to the 1990s but the current nation-wide deworming program for school-age children was only implemented in 2001 [4]. Honduran data from 2003 onwards is available online on WHO's Preventive Chemotherapy (PCT) Databank [19]. Although coverage rates have been sub-optimal, a steady national coverage of around 70% is reported for the last three years for which information is available (2009–2011). Despite this strong commitment to the fight against STH infections, adequate PC monitoring has yet to be implemented in Honduras [20]. Moreover, critical information gaps remain on the prevalence of STH infections as well as on infection intensity and polyparasitism at subnational levels [11].

While it is true that there is a paucity of STH research in Honduras, there is to our knowledge, a wealth of scattered information that has been produced for decades by a number of health professionals, scientists, and students (mainly microbiologists and medical doctors) as well as governmental institutions. Much of this information is, however, not only unpublished but inaccessible to interested stakeholders (*e.g.*, scientific research community, civil society, and decision and policy makers). As shown previously by others, active in-country searches of unpublished data are essential to obtaining epidemiological information to assist in decision-making processes [21]. Therefore, to bring to light this important Honduran information, we carried out a systematic revision of the available published and unpublished literature pertaining STH infections produced in Honduras from 1930 to 2012. Our first objective was to examine the research activity on STH infections, synthesize research evidence, and identify research gaps. Secondly, we selected key data from 2001–2012 to carry out a prevalence analysis for the last decade and map the geographical distribution and prevalence in children ≤15 years old of the three most common intestinal helminths in Honduras, *Ascaris lumbricoides*, *Trichuris trichiura* and the hookworms (predominantly *Necator americanus* [5]).

## Methods

We conducted a scoping review, a method useful for exploring questions where little knowledge has been established [22], [23], and followed the 5-stage methodological framework proposed by Arksey and O'Malley (2005). Briefly, the process included: (1) identifying the research question; (2) identifying relevant studies; (3) study selection; (4) charting the data; and (5) collating, summarizing and reporting the results [24]. We also conducted a stakeholders consultation, previously considered optional [24] but strongly recommended by Levac et al. (2010) to add methodological rigor to these types of studies [23]. Part of the consultation process preceded the literature search and took place in April 2012. The literature search was carried out in Canada and in Honduras from May 2 to June 30, 2012. Analysis and charting of the data was done through an iterative process as defined by Levac et al [23], between July and December 2012.

### Research questions

1. What has been studied in Honduras in regards to STH infections; what are the extent, range, and nature of this research? Derived from these findings, what are the research gaps remaining?
2. What is, for the last decade, the prevalence and geographical distribution of pre-school and school-age STH infections in Honduras? Is STH geographical distribution linked to municipal specific characteristics?

### Literature search strategy

The information search was constrained to the major STH species circulating in Honduras: *Ascaris lumbricoides*, *Trichuris trichiura* and the hookworms. No restrictions in terms of study design, authorship, institutional affiliation, or year of publication were set. For online searches, language was restricted to Spanish and English (the most likely languages to be utilized for international publication by Honduran authors). If review studies were identified, a search of the listed primary sources was done, and if retrieved, they were included in the study.

To identify potentially relevant information (*i.e.*, studies and reports pertaining STH infections in Honduras), we followed the strategy described below.

#### Consultation with government officials, university researchers and other stakeholders

On April 12, 2012, the “Strategic plan for the control, elimination and prevention of neglected infectious diseases in Honduras, 2012–2017” (PEEDH for its Spanish abbreviation) was officially announced in Tegucigalpa. In light of the scarcity of STH research evidence mentioned in PEEDH's inaugural report [25], the need of undertaking the present study became evident. Shortly after, we discussed this collaborative effort with representatives of the Honduran Ministry of Health (MoH) and researchers at the National Autonomous University of Honduras, UNAH. An agreement to share MoH survey data was reached by end of May 2012. Therefore, from June 4–15, 2012, individuals representing the following institutions were approached to discuss their current activities related to STH infections: MoH, World Food Program, Pan American Health Organization (PAHO), Healthy Schools Program, and Parasitology section of the School of Microbiology, UNAH.

#### Online search of internationally published literature

We searched electronic databases including the National Library of Medicine's PubMed (http://www.ncbi.nlm.nih.gov), the Thomson Reuters' Web of Knowledge (http://www.webofknowledge.com) and Google Scholar (http://scholar.google.ca). We also searched databases integrated to the Virtual Health Library, VHL (http://www.bvs.hn) of the Latin American and Caribbean Center on Health Sciences Information (BIREME): LILACS, SciELO, DESASTRES, PAHO, WHOLIS, IBECS and BIMENA.

English terms and their Spanish equivalents were used as keywords. In English, the following keywords were used: soil-transmitted, geohelminth, helminth, *Ascaris lumbricoides*, roundworm, ascariasis, *Trichuris trichiura*, whipworm, hookworm, *Ancylostoma duodenale*, *Necator americanus*, intestinal disease, intestinal obstruction, biliary, diarrhea, parasitism, parasitic, eosinophil, nutrition. Each keyword/term was entered independently into the following search syntax: < keyword or term > AND < Honduras> OR < Honduran >. Each search was done twice on different days to ensure accuracy and maximum return.

#### Online search of literature published in Honduran periodicals

We searched for data available in several online Honduran peer-reviewed periodicals (all affiliated with UNAH, and available at http://www.bvs.hn/RMHS/html/RMH.html). Firstly, we searched Revista Médica Hondureña, (Honduran Medical Journal, established in 1930 and indexed in Medline only until 1965); the most important medium for biomedical research dissemination in Honduras. Secondly, we searched online issues of these more recently established journals: Revista de la Facultad de Ciencias Médicas (est. 2004) Revista Ciencia y Tecnología, UNAH (est. 1997); Revista Médica de los Postgrados de Medicina, UNAH (est. 1996); and Revista Honduras Pediátrica (est.1963 and discontinued 2007). None of the journals supported keyword searches; therefore, potentially relevant articles were identified by screening each available issue.

A newer university journal established in 2011 and devoted to undergraduate and graduate research, Revista Portal de la Ciencia (https://www.unah.edu.hn/?cat=3539), was not included in our search since was not available online. To our knowledge, there are not in Honduras other periodicals -peer-reviewed or otherwise- likely to publish STH research.

For verification, we also searched an online inventory of Honduran national and international publications on intestinal parasites hosted by VHL (http://www.bvs.hn/E/Parasitos.html).

#### Hand search of the grey literature produced locally in Honduras

Grey literature (*i.e.*, studies that are unpublished, have limited distribution, and/or are not included in public bibliographical retrieval systems [26]) was obtained through site visits and meetings with key informants (parasitology faculty members from UNAH, graduate students, government representatives (primarily from the Ministry of Health–some of which took part in the consultation process). From June 4–15, 2012, two libraries affiliated with UNAH, the main Honduran university, were visited to identify potentially relevant research theses and monographs produced by students undertaking professional degrees (*i.e.*, medical sciences, nursing, and microbiology) as well as by graduate students in infectious diseases, public health and epidemiology master in science programs. Prior to visiting the libraries, we found through VHL 72 medical students' theses on intestinal parasites, 69 of which were potentially relevant. Librarians also provided access to library intranets where medical students' monographs had been catalogued. Keyword search was not possible within the intranet so researchers reviewed lists of titles to identify key terms listed above and request access to the documents. Hard copies of theses and monographs selected were retrieved by the librarians for researchers' perusal. Other printed or online material on the subject published by Honduran authors, such as parasitology laboratory manuals and books were scrutinized for primary research data. Key informants were approached to obtain primary data contained in research theses, surveys and field trips reports.

#### Hand search of reference lists on publications by the Pan American Health Organization

Reference lists of PAHO or PAHO-related publications known to the researchers or those found through VHL were screened for primary sources of information.

#### Online searches of STH international databases

Databases of GAHI's Global Atlas of Helminth Infections (http://www.thiswormyworld.org) and the Global Neglected Tropical Diseases (http://www.gntd.org) were searched for Honduran data.

### Selection criteria

All available studies and reports capturing information related to the major STH species circulating in Honduras were assessed by two researchers and two research assistants. Potentially relevant studies were scrutinized and only those providing primary data involving humans and conducted in Honduras were retained for analysis.

### Data extraction

Retained studies were analyzed, charted, and organized into three categories: those reporting clinical cases or investigating medical outcomes (clinical research); those reporting treatment efficacy, and those with an epidemiological scope. Data were entered twice for accuracy into spreadsheets (MS-Excel 2010) and summary tables were produced for each category.

#### Objective 1. Research activity assessment

Research activity was assessed by analyzing published studies in both the peer-reviewed literature and in the form of official reports, theses, and scientific abstracts. Data considered not stemming from research work were excluded from this assessment (*i.e.*, partially processed data derived from parasitology courses field trips and microbiology social service reports summarizing a mandatory one-year professional practice at clinical laboratories of the public health system).

#### Objective 2. Prevalence analysis

Sub-sets of epidemiological studies were utilized to meet the second research objective. Primary data presented by studies were disaggregated into datasets according to date of study, age of population (*i.e.*, children or adult) and outcomes under investigation. Also, as Honduras is organized into 18 departments and 298 municipalities [27], when available, datasets were disaggregated into these first or second administrative levels.

Studies meeting the following criteria were selected for prevalence analysis:

- Providing primary data
- Population- or school-based and dated since 2001, the year national deworming was launched
- Conducted among children (≤15 years old), the target at risk population of PC programs
- Used Kato-Katz for stool examination, the diagnostic method recommended by WHO

Epidemiological studies were reviewed and data extracted into a standardized MS-Excel database. Data extracted included: source of data, author(s), date and location of the study, sample size, characteristics of the surveyed population, parasitological methods performed, and positivity for investigated parasites. Prevalence figures provided by studies were verified or calculated by the researchers conducting the review. Averages of overall and species-specific prevalences were calculated for departmental and municipal levels, and maps were generated to depict their distribution.

### Statistical analysis and prevalence mapping

Descriptive statistics were used to express frequencies. Prevalence of STH infections was calculated from epidemiological studies meeting selection criteria for prevalence analysis. Datasets from surveys done in the same department were averaged to obtain overall prevalence by any STH at the first administrative level. For municipalities, if multiple data were available for the same location, a weighted average prevalence was calculated (accounting for the sample size in each survey). Based on the geographical coordinates of surveys' sites, prevalence maps were generated using ArcGIS 10.1 (ESRI Inc., California, USA). For surveys that had been conducted in small villages nearby municipal capitals, the available geographical coordinates of the latter were used as geo-reference. Prevalence was expressed in risk categories (*i.e.*, low, <20%; moderate, ≥20% and <50%; high, ≥50%), corresponding to WHO's categories of risk for morbidity to determine preventive chemotherapy administration [4].

A multivariable linear regression model was used to assess association between STH prevalence and socio-economic and environmental variables at the municipality level. Socio-economic variables included Human Development Index (HDI), household overcrowding (defined as ≥3 individuals per room, excluding bathrooms and kitchen), households with potable water, and household with sanitary facility [28], [29]. Environmental variables included the average annual temperature and average annual precipitation [30]. Other characteristics considered for analysis were dropped from the model because they were either already included in the HDI calculation (*e.g.*, literacy, unsatisfied basic needs, poverty) or because they showed high collinearity with other variables (*e.g.*, altitude). Statistical analyses were carried out using IBM SPSS Statistics for Windows version 20.0 (Armonk, NY: IBM Corp.) and Stata 13 (College Station, TX: StataCorp LP).

## Results

As initial step, the consultation with stakeholders revealed great interest in harmonizing STH infection control efforts, in particularly the operative aspects of the deworming program. Various stakeholders emphasized the need for monitoring and evaluation of the PC program, while all agreed that integrated, multi-sectoral efforts were needed for STH control to be sustainable. University faculty members highlighted the importance of research capacity strengthening and the need for international collaboration to boost STH research in the country.

The consultation also yielded key data as follows: 3 MoH national surveys [20], [31], [32]; 1 technical report by the Ministry of Natural Resources and Environment [33]; 4 BSc thesis [34]–[37], 1 MPH thesis [38], and 1 PhD thesis [39]. In addition, we obtained 38 datasets with partially processed primary data from parasitology field trips. For a complete list of retained studies and reports, see supporting file Text S1.

**Figure 1:**
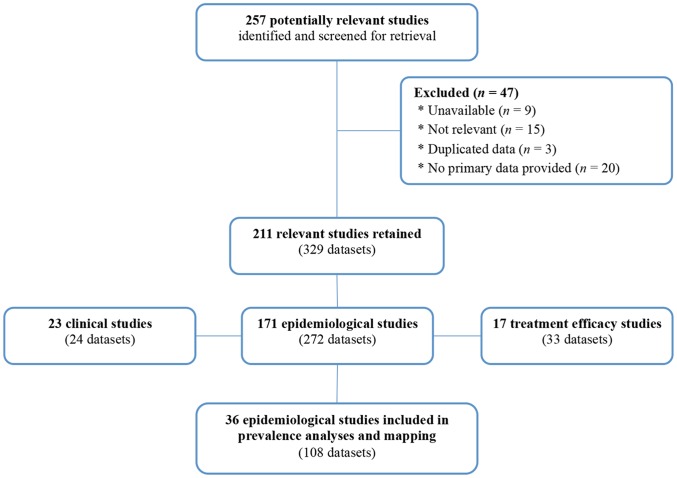
Process of identification and selection of relevant studies (May 1930 to June 30, 2012). Clinical studies included 17 peer-reviewed articles (1 international), 5 medical theses, and 1 conference abstract. Treatment efficacy studies included 6 peer-reviewed articles (3 international), and 11 medical theses. The 36 epidemiological studies used for prevalence mapping included 3 national surveys by the Honduras Ministry of Health, 1 technical report by the Honduras Ministry of Natural Resources and Environment, 28 parasitology courses field trips reports, and 4 undergraduate theses.

On the basis of our consultation efforts and the fact that the Honduran Medical Journal is the oldest Honduran biomedical periodical in which STH-relevant data was likely to be found, our search time period spanned from May 1930, the date this journal was established, to June 30, 2012. A flow chart with details of the literature search and process is shown in Figure 1, and a summary of literature sources and identified, screened, and retained studies is shown in Table 1.

**Table 1:** Summary of identified and screened studies on soil-transmitted helminth infections done in Honduras between May 1930 and June 30, 2012.

Type of source	Source	Online access	Time period searched	N° issues or documents searched	N° potentially relevant studies (No. retained)
Published studies	Honduran journals*	Yes	1930–2011	531	58 (41)
	International journals	Yes	1930–2012	Not applicable	18 (14)**
Grey literature	Medical theses#	No	1950–1992	72	69 (60)
	Medical monographs	No	1993–2012	1140	0 (0)
	Microbiology theses	No	2000–2012	10	6 (6)
	Microbiology monographs	No	2006–2012	89	4 (1)
	Abstracts in scientific conferences	No	1983–2011	28	13 (9)
	Ministry of Health national surveys	No	2001–2011	3	3 (3)
	Other governmental technical reports	No	Not applicable	1	1 (1)
	Microbiology social¥ service reports	No	1990–1992	57	47 (38)
	Parasitology field trips	No	1999–2012	38	38 (38)
	Total				257 (211)

Honduran journals reviewed: 382 issues (from 1930 to 2011) of Revista Médica Hondureña; 20 issues (from 2004 to 2011) of Revista de la Facultad de Ciencias Médicas; 20 issues (from 1997 to 2009) of Revista Ciencia y Tecnología; 29 issues (from 1996 to 2008) of Revista Médica de los Postgrados de Medicina; and 80 issues (from 1963 to 2007) of Revista Honduras Pediátrica.

Of 14 international articles, 1 was in Spanish and 13 in English.

^#^ Catalogued by the Virtual Health Library (Biblioteca Virtual en Salud) as theses done on “Intestinal Parasites” at the Faculty of Medical Sciences (http://www.bvs.hn/E/Parasitos.html).

Other Microbiology Social Service reports had been sent back from the library to the School of Microbiology and discarded by the School due to the lack of storage space.

Searches of international databases were unproductive. GAHI's Global Atlas of Helminth Infections yielded two data sources already identified through our search: data from national surveys and identified on the website as authored by Zúniga, and a paper published internationally [40]. The Global Neglected Tropical Diseases (GNTD) did not contain any data for Honduras. Likewise, searches of PAHO reports or publications [11], [41], [42] did not yield additional primary data.

### Studies identified and selected

**Figure 2:**
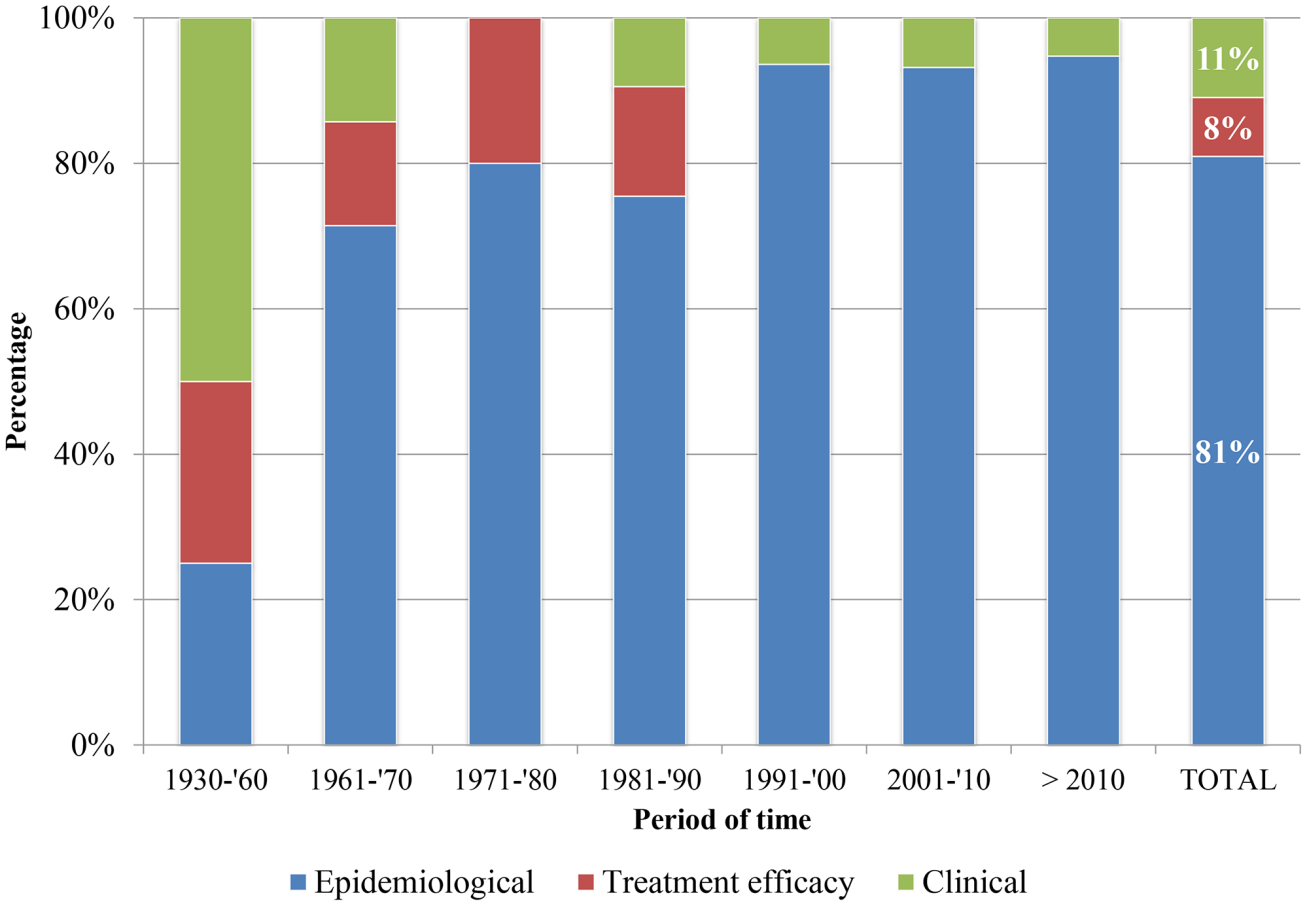
Honduran studies on soil-transmitted helminth infections by category (May 1930 to June 30, 2012). This graph represents 211 studies, either published or in the grey literature, retained for analysis.

As shown in Table 1, of 257 initially identified studies, 76 (29.6%) were scientific peer-reviewed articles published in national or international journals. The remaining 70.4% studies were in the grey literature, most of which only existed in paper on library shelves or researchers' filing cabinets. None of the grey literature was available online. Of the 257 studies selected for scrutiny, 47 (18.3%) were either not found or deemed ineligible. In total, 211 studies comprising 329 datasets were retained. Of 211 studies retained, 23 (10.9%) fell in the clinical research category, 17 (8.1%) focused on treatment efficacy, and 171 (81%) had an epidemiological scope. It was observed that the number of studies on clinical outcomes and treatment efficacy has gradually declined since the 1990s (Figure 2).

### STH research activity in Honduras

**Figure 3:**
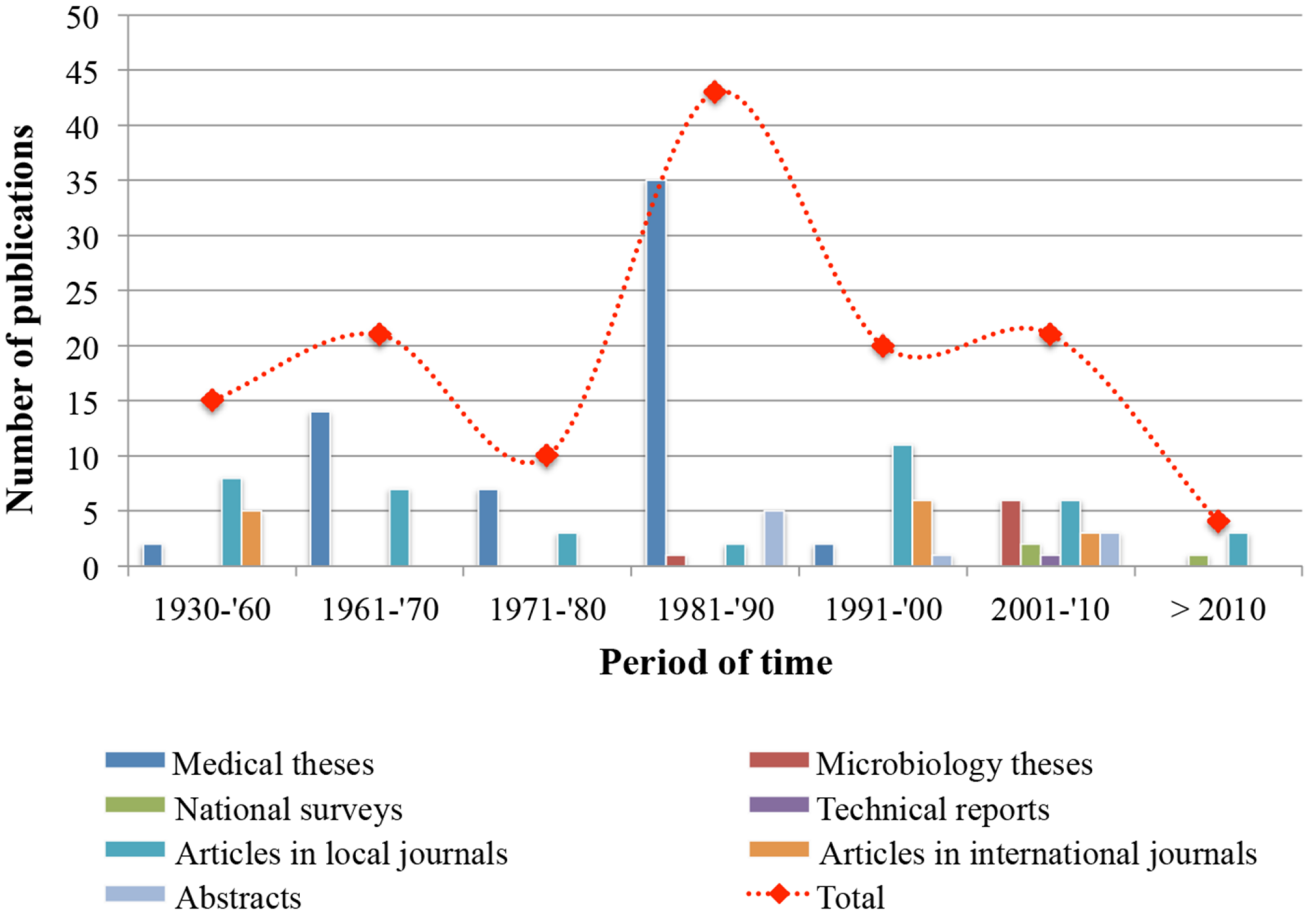
Honduran publication productivity on soil-transmitted helminth infections by source (May 1930 to June 30, 2012). This graph represents 135 documents in both the peer-reviewed and grey literature. Reports from parasitology courses field trips and microbiology social service were excluded from this analysis.

We retrieved a total of 135 publications, 81.5% of which were dated before 2000. The oldest article found containing STH data was published in 1930 [43]. Figure 3 shows an analysis of research productivity by decade and source of publication. Temporal trends were observed in publication activity. Interest in conducting STH research in the faculty of medical sciences is reflected by a 3-decade publications activity. After 1980s, however, a sharp decline in STH publications can be observed. According to library records, no medical theses or monographs related to STH have been written since 1992. Peer-reviewed publications, on the other hand, were more numerous in the late 1950s and again in the 1990s. In the latter decade, 20 publications were produced, 17 of which were articles in Honduran and International journals (of 14 international articles, 13 were in English and one in Spanish. Five were published between 1950 and 1956; six in the 1990s, and three in each 2001, 2002, and 2004. Figure 2 depicts all studies found by category and period of time.

#### Clinical research studies

A summary of 23 STH-related clinical research publications dating from 1932 to 2010 is shown in Table 2. Single-patient or case series were reported by 21 publications (91.3%); the vast majority (n = 17, 80.9%) describing *A. lumbricoides* pathology in children and adults (Table 2A). Four cases involved severe *T. trichiura* pathology (n = 1) or hookworm pathology (n = 3). Altogether, these cases involved 156 patients, nine of whom died. In eight patients, the cause of death was related to *A. lumbricoides* intestinal obstruction or perforation, while in one patient, massive gastrointestinal bleeding due to heavy hookworm infection was reported as the contributory cause. Among these case-reports, special attention should be given to one documenting for the first time the presence of *Ancylostoma duodenale* in Honduras. The case involved a two-month old infant in whom vertical transmission was suspected given the patient's age, exclusive breastfeeding, and maternal hookworm infection [44].

**Table 2:** Clinical studies on soil-transmitted helminth infections published in Honduras from May 1930 to June 30, 2012 (*n* = 23).

A. Case report/Case Series						
Species	Clinical findings	Population	N° reports	N° Cases	Comments	Ref.
*Ascaris lumbricoides*	Biliary ascariasis	Children	3	4	Hepatic abscesses were also found in one case. One deceased.	[75]–[77]
		Adults	5	35	Ultrasound was the most common diagnostic method; only approx. 55% had a positive stool sample	[77]–[81]
	Pleural ascariasis	Children	1	1	Stools were negative for *Ascaris*; secondary to hepatic abscess due to migration of adult worm (Autopsy report)	[82]
	Pseudo-appendicitis	Adults	1	11	Light-moderate chronic abdominal pain was the most frequent symptom.	[83]
	Intestinal obstruction/perforation	Children	4	77	Abdominal pain, vomiting and constipation were the most frequent symptoms. Four deceased.	[84]–[87]
		Adults	3	20	Intestinal perforation was the most frequent complication. Four deceased.	[88]–[90]
*Trichuris trichiura*	Anemia and malnutrition	Children	1	4	Severe anemia and malnutrition were observed due heavy *T. trichiura* infection.	[91]
Hookworms	Anemia	Infant	1	1	Two-month old female patient. Anemia, leukocytosis and eosinophilia. First case of *A. duodenale* reported in Honduras. Vertical transmission suspected.	[44]
	Gastrointestinal bleeding	Infant	1	2	Massive gastrointestinal bleeding due to heavy hookworm infection. One deceased.	[92]
	Anemia	Adult	1	1	Severe anemia and edema in extremities. Heavy infection	[93]

B. Other clinical focus					
Purpose of the study	Population	N° reports	N	Comments	Ref.
Treatment of iron deficiency anemia	Children with anemia	1	25	STH infections were found in 72% of cases	[94]
Determine nutritional status	Adult inmates	1	23	23% of inmates were infected with at least one STH	[95]
	General population	1	198	90% of participants were infected with at least one STH	[45]

STH: Soil-transmitted helminth.

As shown in Table 2B, three clinical research studies (all predating 1970) focused on anemia or nutrition. It is worth highlighting a nutrition study by Borjas (1957), done in a population living in a large area of banana plantations in Northern Honduras. This was a comprehensive collaborative study with the Institute of Nutrition of Central America and Panama (INCAP) and involved an in-depth clinical examination, anthropometric measurements, and laboratory analyses. The study reports a high prevalence of stunting and anemia among children, as well as 90% of STH infection prevalence in the studied population. Almost half of STH infections were caused by multiple parasite species [45].

#### Treatment efficacy studies

Seventeen publications focusing on STH treatment efficacy were found (Table 3). Publication dates ranged from 1954 to 1990. Studies varied greatly in their design but most (73%) were done in children. Altogether, these studies tested the efficacy of 12 different drugs by estimating cure rate (CR), defined as the percentage of individuals who became stool-negative 7–21 days after treatment. Parasite detection in pre- and post-treatment stool samples was done with direct smear (41% of studies), Stoll egg counting technique (41%) or the Kato-Katz method (18%). A pioneer on drug efficacy studies in Honduras was Dr. Mark T. Hoekenga, an American physician who later became the president of the American Society of Tropical Medicine and Hygiene [46]. He tested in humans a variety of drugs against STH and other parasites while stationed in northern Honduras as the Director of Medical Research, United Fruit Company Hospital, La Lima. Hoekenga obtained excellent cure rates for piperazine citrate against *A. lumbricoides* but very poor for hookworms and *T. trichiura* [47]. Albendazole efficacy against all three STH species was reported by two studies in the late 1980s showing CRs ranging from 66% for *T. trichiura* and 100% for hookworms. Conversely, Bustamante and Hernández (1990) studied Albendazole efficacy for hookworms in children, finding a CR of 80% [48].

**Table 3:** Treatment efficacy studies on soil-transmitted helminth infections conducted in Honduras from May 1930 to June 30, 2012 (*n* = 17).

Drug	Dose	Year	Population	*Ascaris*		*Trichuris*		Hookworms		Ref.
				n	CR (%)	n	CR (%)	n	CR (%)	
Albendazole	400 mg (single dose)	1986	General	82	80.5	75	66.7	16	100.0	[96]
		1989	Children	40	90.0	40	92.6	40	100.0	[97]
		1990	Children	—	—	—	—	20	80.0	[48]
Mebendazole	100 mg BID/3 days	1983	Children	21	90.5	18	94.4	4	100.0	[98]
		1986	General	83	78.3	75	65.3	11	100.0	[96]
		1987	Children	45	97.8	—	—	—	—	[99]
		1987	Children	81	44.4	58	70.7	21	9.5	[100]
		1989	Children	37	86.5	37	67.9	37	88.9	[97]
		1989	Children	—	—	7	100.0	4	100.0	[101]
		1990	Children	—	—	—	—	20	90.0	[48]
Piperazine citrate	75 mg/kg BID/2 days	1987	Children	24	62.5	—	—	—	—	[99]
		1989	Children	37	73.0	37	68.8	37	20.0	[97]
	75 mg/kg BID/3 days	1983	Children	21	90.5	20	0.0	1	0.0	[98]
	75 mg/kg BID/7 days	1989	Children	23	87.0	—	—	—	—	[101]
		1989	Children	88	62.5	—	—	—	—	[102]
	100 mg/kg (single dose)	1987	Children	24	58.3	—	—	—	—	[99]
		1989	Children	88	42.0	—	—	—	—	[102]
	1 g BID/6 days	1955	Adults	34	97.0	22	0.0	15	26.4	[47]
	2 g (single dose)/6 days	1955	Adults	51	92.1	52	5.7	33	15.1	[47]
	3 g (single dose)/2 days	1956	Children	30	96.6	—	—	—	—	[103]
Thiabendazole	50 mg/kg BID/3 days	1969	Children	—	—	76	85.5	17	52.9	[104]
		1969	Children			43	27.5	10	80	[105]
Levamisole	3 mg/kg (single dose)	1970	Children	42	97.6	18	44.4	10	80.0	[106]
Pyrantel	10 mg/kg (single dose)	1975	Children	13	92.0	33	8.0	—	—	[107]
		1983	Children	21	100.0	19	0.0	2	100.0	[98]
	20 mg/kg/3 days	1975	Children	—	—	—	—	15	100.0	[107]
Pyrantel-Oxantel	100 mg (single dose)	1987	Children	81	25.9	60	80.0	20	10.0	[99]
Dithiazanine iodide	20 mg/kg/5 days	1960	Children	41	95.0	38	98.0	10	89.0	[108]
	600 mg/5 days	1967	General	—	—	51	72.5	—	—	[109]
Diethylcarbamazine	25 mg/kg/4 days	1954	General	30	80.0	—	—	—	—	[110]
Sodium santoninate	250 mg/6 days	1954	General	38	35.3	—	—	—	—	[110]
Hexylresorcinol	1 g (single dose)	1954	General	80	42.0	—	—	—	—	[110]
Chenopodium-chloroform	15 mL in castor oil	1954	General	80	40.0	—	—	—	—	[110]

#### Epidemiological studies

The majority of studies (171 of 211, 81%) found through our search were epidemiological in nature; they comprised 272 datasets containing coproparasitological results of 342,898 individuals. Thirty-three (19.3%) of these studies were peer-reviewed publications while the rest were unpublished surveys done by either the government or university faculty members. Table 4 shows the characteristics of epidemiological studies included.

**Table 4:** Epidemiological studies on soil-transmitted helminth infections conducted in Honduras from May 1930 to June 30, 2012 (*n* = 171).

Characteristic	*n* (%)
Number of studies identified	171
Peer-reviewed	33 (19.3)
Grey literature	138 (80.7)
Total number of individuals included in the studies	342,898
Studied population	
Children	87 (50.9)
Adults	12 (7.0)
General population	72 (42.1)
Study setting	
Community based	85 (49.7)
Health-care based	73 (42.7)
Special population based	13 (7.6)
Parasite species of focus	
Hookworm only	4 (2.3)
*A. lumbricoides* only	2 (1.2)
All 3 species of STH	165 (96.5)
Primary outcome of the study	
STH prevalence only	140 (81.9)
STH prevalence and risk factors association	10 (5.8)
STH prevalence and pathology association	21 (12.3)
Stool examination method	
Direct smear only (with or without egg counting)	90 (52.6)
Stoll egg counting technique	2 (1.2)
Direct smear and/or Zinc sulphate, sugar flotation, formalin-ether	25 (14.6)
Included Kato-Katz method	54 (31.6)
Intensity of infection	
Established	59 (34.5)
Not established	112 (65.5)

STH: Soil-transmitted helminth.

Prevalence determination and/or infection intensity was the sole objective of 140 studies (81.9%), while 10 (5.8%) and 21 (12.3%) explored associations with either infection risk factors or clinical outcomes, respectively. Half of the studies were conducted in community settings, whereas 42.7% were carried out with hospitalized or outclinic patients in public health care facilities. As shown in Table 4, parasitic infections were determined using various methodologies, mainly the direct smear (52.6%) and Kato-Katz (31.6%). Usage of the Kato-Katz method was traced back to 1993 [49] and by the year 2000, it had become the method of choice. Intensity of infection was assessed in about 34% of the studies. Not all studies using Kato-Katz reported infection intensity. Due to great variability of diagnostic procedures, we did not attempt to analyze infection intensity data.

Ten studies (9 theses and 1 peer-reviewed article) explored risk factors for STH infection. Statistical significance was found between STH infection and low socio-economic status [50], [51] low human development index in the community [39], lack of sanitary infrastructure [50], [52]–[56], and overcrowding [57]. The most recent study examining risk factors for STH in Honduras showed that the number of children in the household with recent history of diarrhea, and infection of household members with *T. trichiura* were associated with ascariasis, and that the number of children 6–14 years old in the household was associated with both *A. lumbricoides* and *T. trichiura* infection [40].

Additionally, two studies reported that older children were more likely to be parasitized with STH than their younger counterparts [56], [58]. Only one study examined the impact of intestinal parasites on school performance [59].

### Prevalence and geographical distribution

Of the 171 epidemiological studies, 36 met the inclusion criteria for prevalence and geographic distribution analysis. We were able to disaggregate these studies by datasets and municipal level and obtained 108 datasets studying 9,336 children from all the country's 18 departments and 69 of the 298 (23%) municipalities. Not all datasets were complete: 12 lacked information on the number of negative samples examined. For this reason, overall STH prevalence was based on only 96 datasets. The majority of municipal datasets derived from single cross-sectional surveys but multiple studies had been done in 18 (26%) municipalities (in which case weighted averages were calculated to estimate prevalence). Also, only 15% of the surveys had been conducted outside municipal capitals. Thus, such data was geo-referenced to the respective capitals' coordinates. Most datasets (70%) were obtained from surveys done by the MoH but additional datasets provided by other authors doubled the sample size and increased the number of municipalities by 15%. Almost half (42%) of the data represented three departments located in central and western Honduras: Francisco Morazán, Santa Bárbara and Copán. Scarcity of data was notorious for departments in the north and east of the country.

Altogether, half (49.1%) of the children studied were positive for at least one STH, and 27.8%, 35.3%, and 5.1% were positive for *A. lumbricoides*, *T. trichiura*, and hookworms, respectively. Consistently, *T. trichiura* prevalence was higher (in average, up to 2.5 times) than that of *A. lumbricoides*.

At the departmental level, *A. lumbricoides* and *T. trichiura* prevalence were above 20% in 16 (89%) departments. Among those, six departments had prevalences of ≥20–50% for both parasites, whereas in two departments (Atlántida and Gracias a Dios) these prevalences exceeded 50%. Hookworm prevalence was more dispersed, ranging from 0–1% and up to 5% in seven and five departments, respectively. The remaining six departments had prevalences ranging from 6% to 23.6%. One Department (Gracias a Dios) had the highest prevalences for all three parasite species: 51.5%, 63.8% and 23.6% for *A. lumbricoides*, *T. trichiura*, and hookworms, respectively.

**Figure 4:**
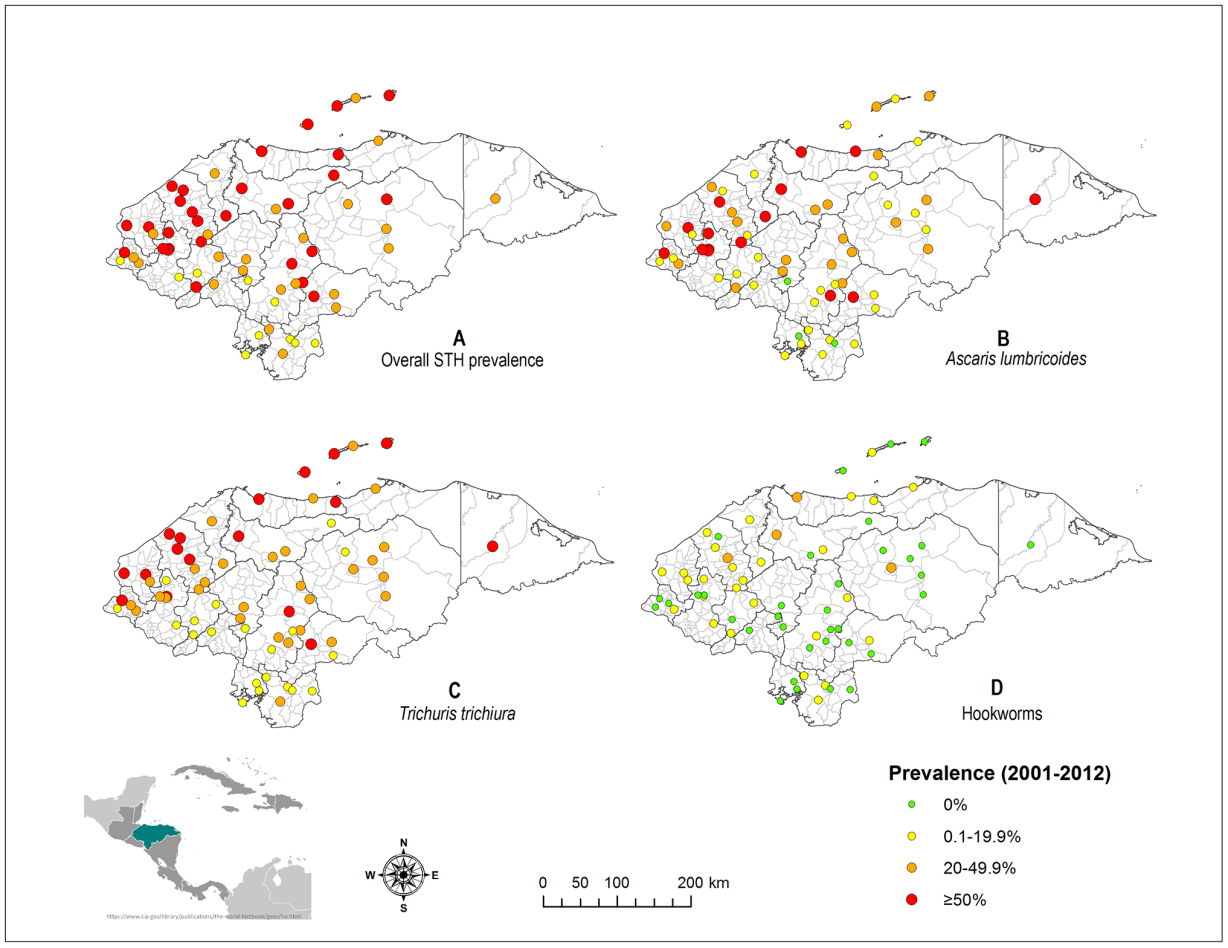
Mapping of observed overall and species-specific prevalence of soil-transmitted helminth infections in Honduras. Data were pooled from 36 epidemiological studies (up to 108 datasets) done between 2001 and 2012. If multiple data were available for the same site (26%), a weighted average prevalence was calculated taking into account the sample size in each survey. Overall prevalence (map A) calculation was done from 96 datasets due to missing data. Prevalences of *A. lumbricoides* (map B), *T. trichiura* (map C), and hookworms (map D), were calculated based on all 108 datasets. Honduras is divided into 18 departments (boundaries defined by black lines on the maps) and 298 municipalities (boundaries defined by gray lines on the maps).

Figure 4 shows the overall and species-specific prevalence as well as the geographic distribution of STH infections according to data from studies done between 2001 and 2012. Map 4A shows that overall STH prevalence was ≥20% in 84% of the municipal data and also that this prevalence was 50% or higher in 40.6% of represented municipalities. As shown in maps 4B and 4C, prevalence data above 20% were common for both *A. lumbricoides* and *T. trichiura* (47.8% and 68% of represented municipalities, respectively). Moderate and high-risk areas for these two parasites overlapped in less than 50% of cases. Hookworm infection was observed throughout the country with prevalence ≥20% in 7.2% of municipalities (Fig. 4D). Half of the study sites had reports of 0% prevalence for hookworm infection. It is important to note that compared with the rest of the country, municipalities in the South, typically with dryer climate and hotter temperature, had consistently lower STH prevalences.

### Prevalence and associated factors

Table 5 shows the results from the multivariable linear regression model testing for associations between selected municipal characteristics and STH prevalence. Human Development Index (HDI) was significantly inversely associated with overall STH prevalence (adjβ = −19.3, 95% CI = −36.12 to −2.48, *p* = 0.025) and *A. lumbricoides* infections (adjβ = −14.2, 95% CI = −27.16 to −1.24, *p* = 0.032). A decrease in about 19% and 14% of overall STH and ascariasis prevalence, respectively, was observed per a 0.1 increase in the HDI value. Likewise, access to potable water in the household was significantly associated with a decrease in the overall STH prevalence (adjβ = −0.57, 95% CI = −1.01 to −0.12, *p* = 0.014). A similar effect, although only marginally significant, was observed for hookworm infections (*p* = 0.069). Conversely, the average annual precipitation was significantly associated with increased overall STH prevalence (adjβ = 0.02, 95% CI = 0.01 to 0.03, *p* = 0.006), as well as with the individual species prevalence [*A. lumbricoides* (adjβ = 0.01, 95% CI = 0.00 to 0.02, *p* = 0.013), *T. trichiura* (adjβ = 0.01, 95% CI = 0.00 to 0.03, *p* = 0.014); hookworm (adjβ = 0.01, 95% CI = 0.00 to 0.01, *p* = 0.023)].

**Table 5:** Association between municipal characteristics and prevalence of soil-transmitted helminth infections in Honduras. 2001–2012.

	Any STH		*A. lumbricoides*		*T. trichiura*		Hookworms	
Municipal characteristic	adjβ (95% CI)	*p*-value	adjβ (95% CI)	*p*-value	adjβ (95% CI)	*p*-value	adjβ (95% CI)	*p*-value
Human Development Index	−19.3 (−36.12, −2.48)	0.025	−14.2 (−27.16, −1.24)	0.032	−10.28 (−26.23, 5.66)	0.202	−2.93 (−8.25, 2.39)	0.275
Household overcrowding	−0.37 (−1.41, 0.67)	0.480	0.04 (−0.74, 0.81)	0.922	−0.34 (−1.17, 0.48)	0.407	−0.08 (−0.38, 0.22)	0.607
Potable water in the household	−0.57 (−1.01, −0.12)	0.014	−0.32 (−0.75, 0.12)	0.147	−0.34 (−0.8, 0.12)	0.144	−0.10 (−0.21, 0.01)	0.069
Sanitary facilities in the household	0.03 (−0.71, 0.76)	0.942	−0.01 (−0.54, 0.53)	0.980	−0.02 (−0.61, 0.58)	0.960	0.08 (−0.13, 0.29)	0.464
Annual precipitation (mm)	0.02 (0.01, 0.03)	0.006	0.01 (0.00, 0.02)	0.013	0.01 (0.00, 0.03)	0.014	0.01 (0.00, 0.01)	0.023
Annual temperature (°C)	−0.27 (−2.86, 2.31)	0.834	−1.79 (−4.07, 0.49)	0.122	0.68 (−1.33, 2.68)	0.501	0.11 (−0.46, 0.68)	0.696

STH, soil-transmitted helminth; adjβ, adjusted coefficient; CI, confidence interval.

## Discussion

The present study provides for the first time, a detailed portrait of past and present efforts to document, research, and define the epidemiological situation of soil transmitted helminthiases in Honduras. This study also provides a 10-year analysis of the geographical distribution and prevalence of STH infections in children ≤15 years of age, the population sector most at risk for these infections. We believe this information is an important first step in understanding the spatial distribution of STH prevalence in Honduras and may assist in the decision-making process in regards to deworming activities or other control and research activities [41], [60].

Other studies have reported that publications on STH infections are scarce in Honduras [11], [41] and our results confirms this to some extent. However, our search identified numerous data sources that had been previously inaccessible to other researchers.

The majority (76%) of publications we found were oriented to either clinical research or case-study reports and were found in the Honduran Medical Journal. Unsurprisingly, these clinical reports tended to describe severe pathology and/or death, which is a common publication bias [61]. Our search also revealed that interest in publishing these types of findings was higher in the 1950s and 1960s, which probably coincided with the heightened attention to tropical medicine in Honduras during that time [46]. Interest in anti-parasitic treatment efficacy was high during the 1950s through the 1970s, but this interest gradually declined in the following decades probably due to the widespread acceptance of benzimidazoles as treatment of choice. Epidemiological studies were found mostly unpublished as the majority were surveys carried out by the government or experiential learning activities in parasitology at a public university.

Our prevalence analysis based on select epidemiological studies shows that most of the Honduran population lives in STH endemic areas. A previous publication provides STH prevalence data for children 1–14 years of age ranging from 11.7%–89.2% [41], while another present an overall national average of >20% [11]. Our study confirms that the prevalence in most of the territory is indeed >20% but more importantly, reveals high-risk geographic areas where STH endemicity is greater than 50%. As pointed out previously [62], spatial distribution analysis is indispensable to understand parasitic disease's transmission dynamics, and the findings presented here provide a valuable insight into Honduras's STH geographical distribution.

The multivariable analysis showed that STH prevalence was significantly associated with lower HDI (a composite index of life expectancy, education, and income). This finding is consistent with previous observations by Ciliézar 2003 [39] and indicates that it is not just poverty what drives STH transmission but other inequities [5]. In addition, higher prevalence of STH infections was observed in municipalities with less access to potable water. Altogether, these findings underscore the importance of integrated approaches to STH control [63]. The overall higher prevalence of *T. trichiura* over *A. lumbricoides* merits further research. The number of infected people globally has been traditionally larger for the latter [18], but it is also well established that benzimidazole drugs' efficacy is significantly lower for *T. trichiura* than for other STH [64]. Due to the annual rounds of single-dose Albendazole currently in place, it is possible that *T. trichiura* will become the predominant species in Honduras. Hence, regular monitoring and research are essential to determine the frequency and schedule of deworming by geographic areas. It also is important to remain vigilant of potential development of drug resistance [14]. In terms of hookworm infections, while the overall national prevalence was scarcely 5.1%, there was great variability not only between municipalities but between studies done in the same location. Overclarification of Kato-Katz preparations leading to loss of hookworm diagnostic reliability could explain, at least partially, this variability. We recently verified this effect during a study in Honduras whereby inadvertent clarification time of >90 minutes reduced Kato-Katz hookworm diagnostic sensitivity by almost 50% [65]. This shows that under-reporting needs to be considered when analyzing national data. It also underscores the need for innovative and standardized diagnostic tools as well as for maintaining adequate training of laboratory personnel [66].

Our study has identified important gaps and challenges in STH research and control in Honduras. Studies on the health, nutrition and cognitive impact of these helminthiases in children are greatly needed. Equally important are studies that accurately determine prevalence and infection intensity by ecological zones along with associated risk factors for infection. Research gaps were also revealed around benzimidazoles efficacy. From the policy and practice perspective, an important challenge at present is the lack of a monitoring and evaluation (M&E) component to the PC program. If implemented, M&E performance indicators would be useful in tracking progress towards the morbidity target recommended by the WHO (*i.e.*, reduce STH infections of moderate and high intensity below 1%) [4].

### Strengths and limitations

Major strengths of this study are its breadth and depth. We made every possible effort to identify, locate, and retrieve a vast amount of work and data from both published and unpublished sources, thereby reducing many of the well-known biases that affect systematic review studies [67]. Importantly, by conducting our search in two languages and not excluding the grey literature in our analysis, we reduced biases in location [67], selective publication [68] and dissemination [61]. Almost certainly, however, there may remain many other studies or data sources that we were not able to identify (*e.g.*, from non-governmental organizations providing deworming). As well, our prevalence analysis has unavoidable limitations inherent to pooling of historical data, as indicated in recent publications [69], [70]. Aware of the diverse quality of studies included (in terms of sampling strategies and diagnosis accuracy [71], [72]), we aimed at reducing bias by selecting within the epidemiological studies those that were less dissimilar, namely, using data gathered from 2001 onwards, avoiding pooling data from different age groups, and restricting provenance of studies to community and school-based only. Further, to mitigate diagnostic differences, we only selected studies utilizing single-stool examination by the Kato-Katz method. Still, it is widely known that Kato-Katz is subject to variations, and while our prevalence data for *A. lumbricoides* and *T. trichiura* are probably reliable, the same cannot be said for hookworm data.

Limitations notwithstanding, we provide further evidence that STH infections are widespread in Honduras and are likely affecting the quality of life of the poorest of the poor [3], [73]. Moreover, we reveal prevalence differentials at sub-national administrative levels, which can prove useful for establishing a historical trail of these infections, and most importantly help design better interventions for their prevention and control [60].

### Five-year outlook and conclusions

Honduras is making good strives toward sustained implementation of a nation-wide deworming program of both pre-school and school-age children. Here, we propose a five-year outlook of the types of strategies and activities that Honduras as a country could prioritize (Table 6). Implementing such strategies would help Honduras not only overcome current challenges for sustainable control but also to fill the knowledge gaps identified in the present study.

**Table 6:** A five-year outlook of strategies that could be implemented in Honduras to achieve sustainable control of soil-transmitted helminth infections.

Strategy	Description
Integrated, inter-sectoral interventions	Apply collaborative, integrated and multi-sectoral approaches especially prioritizing communities where focal transmission shows singular patterns
	Evaluate the feasibility of deploying complementary interventions such as improved sanitation, vector control, and health promotion
	Integrate communities and municipalities to play a bigger role in sanitation, health education, and treatment uptake
Regular evaluation and monitoring of control efforts	Studies to monitor and evaluate the performance and reach of the national soil-transmitted helminth infections control program
Fill critical clinical knowledge gaps	Studies around benzimidazole efficacy for the three soil-transmitted parasites species prevalent in Honduras
	Research into the co-management of these and other diseases
Expand epidemiology research efforts	Studies on the health, nutrition and cognitive impact of these helminthiases in children
	Determine prevalence and infection intensity by ecological zones along with associated risk factors for infection
	Update the mapping of STH prevalence and intensity, including estimates of populations at risk and disease burden
Research capacity strengthening	Reinforce the training and capacity of medical, laboratory, and other health professionals
	Enhance statistical analysis capability including geospatial mapping
	Seek international research collaboration
Knowledge transfer and dissemination	Improve coordination and information dissemination to gain local and international financial and political support
	Disseminate research findings to local and international stakeholders using appropriate outlets
	Share information with the scientific community through peer-reviewed journals and other accessible media

In conclusion, we believe the way forward is to increase the amount and quality of STH research done in Honduras and to make this knowledge publicly available to the scientific community as well as to national and international policy and decision makers. At the same time, efforts directed to STH control and prevention should continue with firm determination and more diversification. As PC coverage reaches the national goal, we advocate for integrated, multi-sectoral approaches [5], [63], [74] prioritizing communities where focal transmission shows singular patterns. Differential endemicity is possible because even though Honduras is a small country (112,492 km^2^); it is characterized by a mountainous territory comprising distinct ecological areas with important variations in precipitation, temperature, soil composition, etc. It is also a nation of diverse cultures and ethnicities, and with profound social inequalities [28]. Ultimately, with strong support from the national and international community, Honduras's strategic plan for NTD control has the potential to greatly reduce the health burden of STH infections and other neglected diseases in the country.

## Supporting Information

Table S1 PRISMA checklist.
(DOC)

Text S1 Studies retained for analysis.
(PDF)
