# Supplementary material for: A Scoping Review and Prevalence Analysis of Soil-Transmitted Helminth Infections in Honduras
Source: PLoS Negl Trop Dis. 2014 Jan 23;8(1):e2653. doi: 10.1371/journal.pntd.0002653 (PMC3900402; doi:10.1371/journal.pntd.0002653)
Supplement: Text S1 — Studies retained for analysis. (PDF) [file pntd.0002653.s002.pdf]

A scoping review and prevalence analysis of soil-transmitted helminth infections in Honduras

Text S1: Studies retained for analysis

A. Peer-reviewed

A.1 Honduran journals*

1. Arima Y, Kaminsky RG, A. AG, Casiano-Colón A, Guthrie BL, et al. (2011) [New and old agents of acute diarrhea in children in Honduras]. Revista Médica Hondureña 79: 58-64. (Article in Spanish).

2. Bendeck AC (1965) [Iron-deficiency anemias of diverse etiologies treated with intramuscular iron preparation]. Revista Honduras Pediátrica 1: 11-21. (Article in Spanish).

3. Bendeck AC, Argeles P, Aguilera R, Durón L, Jirón J, et al. (1969) [Studies on diarrhea in Honduras]. Revista Honduras Pediátrica 3: 296-315. (Article in Spanish).

4. Bendeck AC, Barahona GA, Atuán J, Stefan R, Sandoval R (1970) [Thiabendazole in the treatment of trichuriasis and hookworm infections]. Revista Honduras Pediátrica 4: 128-142. (Article in Spanish).

5. Bendeck AC, Larios ME, Farach VZ (1983) [Study of the etiology of childhood diarrhea in Honduras]. Revista Honduras Pediátrica 9: 4-10. (Article in Spanish).

6. Borjas EA (1954) [Nutrition study in inmates in the Central Penitentiary, Tegucigalpa]. Revista Médica Hondureña 22: 663-669. (Article in Spanish).

7. Borjas EA (1957) [Nutrition studies in Honduras]. Revista Médica Hondureña 25: 4-17. (Article in Spanish).

8. Cáceres M (1934) [Hookworm infection in Honduras]. Revista Médica Hondureña A4: 323-324. (Article in Spanish).

9. Castro-Gonzales F (2001) [*Ascaris lumbricoides* complications in hospitalized children, Hospital Escuela, Honduras]. Revista Médica Postgrado 6: 291-298. (Article in Spanish).

10. Dala FE, Cleaves F, Velásquez O, López MM, Zavala A (1991) [Massive digestive bleeding by hookworm infection: presentation of two clinical cases]. Revista Honduras Pediátrica 14. (Article in Spanish).

11. Durón RA (1962) [Pleural ascariasis]. Revista Médica Hondureña 30. (Article in Spanish).

12. Fajardo DA, Toledo ML, López M (2003) [Cholecystitis and hepatic granulomatous inflammation secondary to *Ascaris lumbricoides* eggs]. Revista Médica Hondureña 71: 18. (Article in Spanish).

13. Galeas-Castillo B, Durón I (1998) [Ascariasis in biliary tract, Hospital Escuela, Honduras]. Revista Médica Postgrado 5: 90-99. (Article in Spanish).

14. García T, Barrientos JA, Bonilla C (1975) [Pyrantel pamoate for intestinal parasitic diseases]. Revista Honduras Pediátrica 6: 623-629. (Article in Spanish).

15. Gómez-Márquez J (1952) [Ascariasis as ecological factor for intestinal obstruction in children]. Revista Médica Hondureña 20: 95-97. (Article in Spanish).

16. Gutiérrez R (1955) [Intestinal perforations caused by parasites]. Revista Médica Hondureña 23: 805-817. (Article in Spanish).

17. Kaminsky RG (1997) [Intestinal parasites in different populations in Honduras]. Revista Médica Hondureña 65: 118-119. (Article in Spanish).

18. Kaminsky RG (1999) [Intestinal parasites in different populations in Honduras: III. Prevalence of intestinal parasites in HIV/AIDS patients]. Revista Médica Hondureña 67: 235-242. (Article in Spanish).

19. Kaminsky RG (2000) [Intestinal parasites in different populations in Honduras: IV. Commercial sex workers]. Revista Médica Hondureña 68: 134-140. (Article in Spanish).

20. Kaminsky RG (2000) [First report of *Ancylostoma duodenale* in Honduras: clinical and parasitological description]. Revista Médica Hondureña 68: 142-148. (Article in Spanish).

21. Kaminsky RG (2002) [Statistical update of intestinal parasitism. Laboratory results, Hospital Escuela, Honduras]. Revista Médica Hondureña 70: 57-69. (Article in Spanish).

22. Kaminsky RG, Canales M (1986) [Cryptosporidiasis in children under six years of age with gastroenteritis in Honduras]. Revista Médica Hondureña 54: 268-277. (Article in Spanish).

23. Kaminsky RG, Flores R, Alberto S, Milla V (1998) [Prevalence of intestinal parasites in different populations in Honduras: II. Children and adults in institutions]. Revista Médica Hondureña 66: 62-70. (Article in Spanish).

24. Kaminsky RG, Javier A, Castillo V (2000) [Helminth prevalence in children, municipality of Santa Ana, Honduras]. Revista Honduras Pediátrica 21. (Article in Spanish).

25. Kaminsky RG, Lupiac JA (2011) [Strongyloidiasis, taeniasis and other neglected parasitic diseases in prisoners, Honduras]. Revista Médica Hondureña 79: 122-127. (Article in Spanish).

26. Kaminsky RG, Pineda RZ, Ordóñez E, Valenzuela R (2010) [Neglected parasitosis: severe trichuriasis in hospitalized children, Hospital Escuela, Honduras]. Revista de la Facultad de Ciencias Médicas 7: 37-38. (Article in Spanish).

27. Kaminsky RG, Retes EH (2000) [Helminthiases in children from Amapala, Honduras]. Revista Honduras Pediátrica 21. (Article in Spanish).

28. Kaminsky RG, Stovall ME, Mayer ML, Martin AD, Bowers LC, et al. (2007) [Intestinal microsporidiosis in AIDS patients from Honduras]. Revista Médica Hondureña 75: 116-123. (Article in Spanish).

29. Landa L, Vijil M (1991) [Intestinal parasitism in childhood]. Revista Médica Hondureña 59: 87-90. (Article in Spanish).

30. Larios M (1930) [Amebiasis: incidence and diagnosis of amebiasis in Tegucigalpa]. Revista Médica Hondureña A0: 3-12. (Article in Spanish).

31. Matamoros B (1960) [Report on the use of a broad-spectrum anthelminthic, La Lima Hospital]. Revista Médica Hondureña 28. (Article in Spanish).

32. Murillo E, González AL (2011) [Hepatobiliary ascariasis with abscesses: a case report from Honduras]. Revista Médica Hondureña 79: 167-170. (Article in Spanish).

33. Sánchez AL, Reyes T, Ljungström I, Medina MT (1996) [Epidemiological study of taeniasis and cysticercosis in Honduras]. Revista Médica Hondureña 64: 47-51. (Article in Spanish).

34. Sorto R, Bú-Figueroa E (2006) [Clinic and parasitological profile of HIV/AIDS patients treated in the Hospital Escuela during 2003-2005 period]. Revista Médica Hondureña 74: 69-76. (Article in Spanish).

35. Tábora JE (1960) [Frequency of intestinal parasitism in patients attending the maternal/child clinic "El Manchén"]. Revista Honduras Pediátrica 2: 32-40. (Article in Spanish).

36. Zepeda JE (1972) [Copro-parasitological study in three primary schools in sub-urban Tegucigalpa, Honduras]. Revista Médica Hondureña 40: 119-123. (Article in Spanish).

37. Zepeda JE, Barahona GA (1970) [Copro-parasitological study in school children from El Chimbo, Francisco Morazán, Honduras]. Revista Médica Hondureña 38: 195-198. (Article in Spanish).

38. Zepeda JE, Barahona GA (1970) [Copro-parasitological study in school children from Santa Lucía, Francisco Morazán, Honduras]. Revista Médica Hondureña 38: 74-77. (Article in Spanish).

39. Zepeda JE, Barahona GA (1971) [Copro-parasitological study in school children from Monjarás, Choluteca, Honduras]. Revista Médica Hondureña 39: 306-309. (Article in Spanish).

40. Zúñiga SR, Banegas V (1966) [Acute pseudoapendicitis]. Revista Médica Hondureña 34: 15-30. (Article in Spanish).

41. Zúñiga SR, Gómez-Márquez J, Vargas AD (1960) [Biliary ascariasis: presentation of three cases]. Revista Médica Hondureña 28: 132-145. (Article in Spanish).

**Note: Free translation made by the review authors for publications in Revista Médica Hondureña before 1990, and for publications in any other Honduran journal.*

A.2 International journals

42. Espinoza LM, Soto RJ, Alger J (1999) [Eosinophilia associated to helminthiases in children attending Hospital Escuela, Honduras]. Revista Mexicana de Patología Clínica 46: 79-85. (Article in Spanish).

43. Hoekenga MT (1950) The incidence of human intestinal parasites in northwestern Honduras. The American Journal of Tropical Medicine and Hygiene 30: 757-759.

44. Hoekenga MT (1954) Experiments in the therapy of human ascariasis. Am J Trop Med Hyg 3: 755-761.

45. Hoekenga MT (1955) Treatment of multiple intestinal worm infections with piperazine citrate. The American Journal of Tropical Medicine and Hygiene 4: 1088-1090.

46. Hoekenga MT (1956) Experiments in the therapy of human ascariasis, with particular reference to the piperazine salts. World Med J 3: 279-283.

47. Kaminsky RG (1991) Parasitism and diarrhoea in children from two rural communities and marginal barrio in Honduras. Transactions of the Royal Society of Tropical Medicine and Hygiene 85: 70-73.

48. Kaminsky RG, Soto RJ, Campa A, Baum MK (2004) Intestinal parasitic infections and eosinophilia in an human immunedeficiency virus positive population in Honduras. Memorias do Instituto Oswaldo Cruz 99: 773-778.

49. Kwa BH, Aviles R, Tucker MS, Sanchez JA, Isaza MG, et al. (2004) Surveillance for enteric parasites among U.S. military personnel and civilian staff on Joint Task Force Base-Bravo in

Soto Cano, Honduras and the local population in Comayagua and La Paz, Honduras. Military Medicine 169: 903-909.

50. Lindo JF, Dubon JM, Ager AL, Gourville EM, Solo-Gabriele H, et al. (1998) Intestinal parasitic infections in human immunodeficiency virus (HIV)-positive and HIV-negative individuals in San Pedro Sula, Honduras. The American Journal of Tropical Medicine and Hygiene 58: 431-435.

51. Lozano RH (1955) *Ascaris lumbricoides* and obstructive jaundice: report of a case. J Int Coll Surg 23: 724-728.

52. Sanchez AL, Gomez O, Allebeck P, Cosenza H, Ljungstrom L (1997) Epidemiological study of *Taenia solium* infections in a rural village in Honduras. Ann Trop Med Parasitol 91: 163-171.

53. Sanchez AL, Lindback J, Schantz PM, Sone M, Sakai H, et al. (1999) A population-based, case-control study of *Taenia solium* taeniasis and cysticercosis. Ann Trop Med Parasitol 93: 247-258.

54. Sanchez AL, Medina MT, Ljungström I (1998) Prevalence of taeniasis and cysticercosis in a population of urban residence in Honduras. Acta Trop 69: 141-149.

55. Smith H, Kaminsky RG, Niwas S, Soto R, Jolly P (2001) Prevalence and intensity of infections of *Ascaris lumbricoides* and *Trichuris trichiura* and associated socio-demographic variables in four rural Honduran communities. Mem Inst Oswaldo Cruz 96: 303-314.

B. <u>Grey literature</u>

B.1 Medical students' theses

56. Aguilar CR (1988) Estudio clínico terapeutico experimental entre Mebendazol y Oxantel Pirantel en la población pediátrica del Hospital Santa Bárbara Integrado. Octubre-Noviembre, 1987. Tegucigalpa, MDC: UNAH. 53 p.

57. Alvarado JS (1991) Parasitismo intestinal en niños desnutridos menores de 5 años, hospitalizados en el Centro Nutricional Santa Ana de El Negrito, Yoro. Diciembre 1990 a Marzo 1991. Tegucigalpa, MDC: UNAH. 75 p.

58. Antúnez HG (1985) Enfoque integral preventivo del parasitismo intestinal en la comunidad de Marale, Francisco Morazán. Tegucigalpa, MDC: UNAH. 94 p.

59. Ayes FE (1986) Obstrucción intestinal por *Ascaris lumbricoides* en el Hospital Tela Integrado. Tegucigalpa, MDC: UNAH. 161 p.

60. Bulnes NZ, López CD (1990) Estudio de la prevalencia de uncinariasis y sus determinantes socio-económicos y culturales en la población mayor de 7 años de la aldea de El Bijao, Olancho, 1989 Honduras, C.A. Tegucigalpa, MDC: UNAH. 125 p.

61. Bustamante AR, Hernández HE (1990) Prevalencia de uncinariasis y eficacia del tratamiento con Albendazol y Mebendazol en niños de edad escolar en los departamentos de Colón, Cortés y Ocotepeque. Tegucigalpa, MDC: UNAH. 62 p.

62. Caballero W (1983) Un estudio comparativo de tres drogas antiparasitarias en la población escolar del municipio de Jesús de Otoro, en el departamento de Intibucá. Tegucigalpa, MDC: UNAH. 101 p.

63. Carías M (1962) Breve estudio sobre parasitismo intestinal. Tegucigalpa, MDC: UNAH.27 p.

64. Cerrato NC (1990) Parasitismo intestinal en poblacion escolar. Estudio de intervención. Dulce Nombre de Copán. Tegucigalpa, MDC: UNAH. 100 p.

65. Chaín HR (1989) Helmintiasis intestinal confirmada laboratorialmente y terapéutica de la ascariasis intestinal a base de la Piperazina en pacientes comprendidos entre la edad de 0-15 años en la aldea de la Cumbre, municipio de la Masica, Atlántida, durante el programa de desparasitación en el mes de Enero de 1989. Tegucigalpa, MDC: UNAH. 78 p.

66. Chang HR (1984) Parasitismo intestinal, uncinariasis, en el municipio de Gualala, Santa Bárbara, 1981. Honduras C.A. Tegucigalpa, MDC: UNAH. 58 p.

67. Chávez E, Elvir MJ (1980) Algunos indicadores del estado de salud de la población escolar de Olanchito. Tegucigalpa, MDC: UNAH. 41 p.

68. Cruz D (1979) Prevalencia de la parasitosis intestinal en la comunidad de Cofradía, Cortés y su relación con otros lugares del país. Tegucigalpa, MDC: UNAH. 35 p.

69. Cubas RF (1982) Prevalencia del parasitismo intestinal en la población menor de seis años de edad en la comunidad de la Esperanza, Intibucá. Tegucigalpa, MDC: UNAH.

70. Del Cid E (1988) Estudio de intervención con charlas educativas a familiares de niños parasitados y tratamiento comparativo. Tegucigalpa, MDC: UNAH. 78 p.

71. Elvir MN (1988) Obstrucción intestinal en niños de 0-13 años de edad. Hospital Leonardo Martínez V. 1984-1987. Tegucigalpa, MDC: UNAH. 130 p.

72. Enamorado E (1966) Aspecto epidemiológico de la uncinariasis en el distrito sanitario nº 3. Tegucigalpa, MDC: UNAH. 40 p.

73. Gallardo MH (1985) Incidencia de algunos parásitos intestinales en niños de 0-5 años con síndrome diarreico agudo observado en la consulta externa del Centro Hospitalario de Emergencia del municipio de Tocoa, departamento de Colón. Tegucigalpa, MDC: UNAH. 79 p.

74. Gutiérrez RE (1985) Incidencia de parasitosis intestinal en niños de ambos sexos, menores de cinco años, en el Hospital Dr. Salvador Paredes en Trujillo, departamento de Colón. Tegucigalpa, MDC: UNAH. 120 p.

75. Handal EA (1976) Levamisol en el tratamiento de ascariasis, uncinaria y tricocéfalos. Tegucigalpa, MDC: UNAH. 53 p.

76. Hernández FA (1967) Parasitismo intestinal en Santa Rosa de Copán. Tegucigalpa, MDC: UNAH. 38 p.

77. Interiano P (1966) Uncinariasis. Tegucigalpa, MDC: UNAH. 22 p.

78. Johnson J (1968) Parasitismo intestinal en el Hospital General y Asilo de Inválidos. Tegucigalpa, MDC: UNAH. 37 p.

79. Lagos W (1979) Parasitismo intestinal: sus alteraciones hematológicas y nutricionales. Estudio realizado en la comunidad de Ajuterique, departamento de Comayagua. Tegucigalpa, MDC: UNAH. 40 p.

80. Lizardo JA (1984) Obstrucción intestinal mecánica en el Hospital del Sur. Tegucigalpa, MDC: UNAH. 67 p.

81. López C (1968) Nemátodos intestinales en la sala de pediatría en el Hospital de Occidente de Santa Rosa de Copán. Tegucigalpa, MDC: UNAH. 16 p.

82. Maradiaga A (1969) Uncinariasis: su tratamiento con Tiabendazol. Tegucigalpa, MDC: UNAH. 48 p.

83. Maradiaga EE (1982) Prevalencia de parasitosis intestinal en la sala de hidratación oral del Hospital Escuela. Tegucigalpa, MDC: UNAH. 46 p.

84. Maradiaga IM (1982) Estudio sobre parasitismo intestinal en niños de la Guardería Infantil San Isidro. Comayagüela, D.C. Tegucigalpa, MDC: UNAH. 47 p.

85. Martínez HE (1987) Multiparasitismo intestinal asociado a desnutrición en la población infantil escolar menor de 12 años en la ciudad de Yoro, Yoro. 1986. Tegucigalpa, MDC: UNAH. 95 p.

86. Matute RA (1966) Uncinariasis en el departamento de Santa Bárbara: (Estudio hecho en el Hospital de Santa Bárbara). Tegucigalpa, MDC: UNAH. 29 p.

87. Medina AE (1988) Albendazol y Piperazina en el tratamiento de ascariasis intestinal. Tegucigalpa, MDC: UNAH. 80 p.

88. Medina L (1981) Estudio de la prevalencia de parasitismo intestinal en la población menor de 6 años de la comunidad de Catacamas, Olancho. Tegucigalpa, MDC: UNAH. 30 p.

89. Miralda LR (1982) Estudio descriptivo de helmintiasis intestinal en el Hospital Manuel de Jesús Subirana. Tegucigalpa, MDC: UNAH. 78 p.

90. Morales JS (1981) Parasitosis intestinal, prevalencia a nivel de la comunidad de El Progreso, Yoro y su relación con otros lugares del país. Tegucigalpa, MDC: UNAH. 81 p.

91. Murillo F (1959) Abdomen quirúrgico por parasitismo intestinal, relación de 15 casos presentados en el Hospital General San Felipe. Tegucigalpa, MDC: UNAH. 51 p.

92. Naranjo LF (1987) Parasitismo intestinal en población escolar. Estudio de intervención, Teupasenti, El Paraíso. Tegucigalpa, MDC: UNAH. 121 p.

93. Navarro LA (1982) Estudio sobre helmintiasis intestinal realizado en la población infantil de la comunidad de Siguatepeque, depto. de Comayagua. Tegucigalpa, MDC: UNAH. 43 p.

94. Núñez NG (1990) Incidencia de ascariasis biliar en el Hospital Escuela en el período de Enero de 1980 a Diciembre de 1989. Tegucigalpa, MDC: UNAH. 84 p.

95. Núñez VL (1990) Parasitismo intestinal en el municipio de Guata, departamento de Olancho: su relación con algunas condiciones de vida y su efecto sobre índice peso-talla en escolares. Tegucigalpa, MDC: UNAH. 52 p.

96. Ortega LA (1967) Parasitismo intestinal en la ciudad de El Paraíso. Tegucigalpa, MDC: UNAH. 27 p.

97. Pérez JA (1992) Estudio de *Entamoeba histolytica*, *Ascaris lumbricoides* y *Trichuris trichiura* en el casco urbano de La Libertad, Comayagua. Tegucigalpa, MDC: UNAH. 51 p.

98. Pérez JM, Matamoros H (1982) Estudio sobre uncinariasis en Monjarás, Choluteca, 1982. Honduras, C.A. Tegucigalpa, MDC: UNAH. 104 p.

99. Pineda H (1960) El parasitismo intestinal en Amapala. Tegucigalpa, MDC: UNAH. 44 p.

100. Pineda JA (1986) Ensayo clínico terapeútico comparando el Albendazol y el Mebendazol en el tratamiento de las helmintiasis intestinales realizado en Santa Rosa de Copán. Enero y Febrero de 1986. Tegucigalpa, MDC: UNAH. 91 p.

101. Pineda N (1978) Estudio de la prevalencia de parasitosis intestinal en la población escolar de la comunidad de Minas de Oro. Tegucigalpa, MDC: UNAH. 37 p.

102. Ramírez DM (1973) Efectos de la labor del equipo de enfermería en la prevención y control de las parasitosis intestinales de pacientes que asisten al consultorio del subcentro de salud Dr. M.G. Zúniga, Tela, Atlántida, Honduras C.A. 1972. Tegucigalpa, MDC: UNAH. 91 p.

103. Ramos F (1974) Características y recursos de salud y la incidencia de parasitosis intestinal en el Hospital San Francisco. Tegucigalpa, MDC: UNAH. 69 p.

104. Renderos JI, Torres RF, Meléndez AE (1989) El parasitismo intestinal y su respuesta a la terapia antiparasitaria convencional en niños desnutridos atendidos en el CESAMO y SERN (Servicio de Educación y Recuperación Nutricional) de Olanchito. Tegucigalpa, MDC: UNAH. 91 p.

105. Reyes C (1965) La uncinariasis como problema sanitario en Honduras. Tegucigalpa, MDC: UNAH. 34 p.

106. Ríos GA (1988) Ascariasis en adultos y su relación con el estado nutricional en la comunidad de Yoro, 1988. Tegucigalpa, MDC: UNAH. 77 p.

107. Rivas LG (1967) Incidencia de parasitosis intestinal registrada en el Hospital San Francisco de Juticalpa, depto. de Olancho, durante los años de 1963-66. Tegucigalpa, MDC: UNAH.

108. Rivera P (1966) La uncinariasis como problema de salud en Honduras. Tegucigalpa, MDC: UNAH. 24 p.

109. Rodríguez AR (1968) Tricocefalosis y terapia con Yoduro de Ditiazaminal (Netocyd). Tegucigalpa, MDC: UNAH.

110. Rubio JA (1981) Estudio de ciento cuarenta y seis casos de anemia en el Hospital San Francisco de Juticalpa, Olancho. Tegucigalpa, MDC: UNAH.

111. Sánchez R (1989) Población escolar del Cajón, su parasitismo infantil y niveles de hemoglobina, hematocrito, proteínas totales, albúminas, globulinas (estudio 82 casos). Tegucigalpa, MDC: UNAH. 130 p.

112. Sandoval O, Rodríguez EP (1986) Estudio de la incidencia y características del parasitismo intestinal en niños en edad escolar en la comunidad de Tocoa, Colón 1986. Tegucigalpa, MDC: UNAH. 160 p.

113. Stefan R (1970) Tiabendazole en el tratamiento de tricocefalosis y uncinariasis. Hospital Materno Infantil. Tegucigalpa, MDC: UNAH. 57 p.

114. Valle JA, López DA, Lama SG (1985) Parásitos, rotavirus y bacterias enteropatógenas presentes antes, durante y después de un episodio diarreico en niños menores de 2 años en la ciudad de Siguatepeque, Comayagua. Año 1984-1985. Tegucigalpa, MDC: UNAH. 121 p.

115. Velásquez LA (1989) Estudio comparativo del Albendazol, Mebendazol y Piperacina en el tratamiento de la helmintiasis intestinal en pacientes mayores de dos años, ingresados durante un período de tres meses a la sala de pediatría del Hospital Regional Atlántida. Mayo - Julio 1989. Tegucigalpa, MDC: UNAH. 74 p.

B.2 Microbiology students' theses

116. Cáceres R (2009) Estudio parasitológico en población escolar de 6-14 años del Rodeo, Jute, Cajón y Tierra Chela [BSc.]. Tegucigalpa, MDC: UNAH.

117. Canales M (2008) Factores de riesgo asociados a la transmisión de teniasis cisticercosis, en la aldea de Jalaca, una comunidad rural de Francisco Morazán. Honduras. 2001-2002 [MPH.]. Tegucigalpa, MDC: UNAH.

118. Ciliezar A (2003) Hacia una estrategia social integral del control de parásitos [PhD.]. Tegucigalpa, MDC: UNAH.

119. Ferrera A (1987) Influencia climática sobre algunos helmintos en las comunidades de Tegucigalpa y Choluteca [BSc.]. Tegucigalpa, MDC.: UNAH.

120. García JA (2008) Prevalencia de helmintos transmitidos por el suelo en niños de edad escolar del municipio de Macuelizo, Santa Bárbara [BSc.]. Tegucigalpa, MDC: UNAH. 87 p.

121. Mejía JG (2010) Diagnóstico de parásitos intestinales en estudiantes universitarios [BSc.]. Tegucigalpa: UNAH.

122. Sosa WH (2007) Relación entre infecciones de helmintos transmitidos por el suelo y anemia en niños escolares durante pre y post tratamiento con antihelmíntico y suplemento vitamínico, en el valle de Macuelizo, departamento de Santa Bárbara, Honduras. [BSc.] Tegucigalpa, MDC: UNAH. 64 p.

B.3 Abstracts in scientific conferences

123. Canales M, Thumman L. Estudio de parásitos intestinales en niños de 1-15 años de edad en dos barrios marginales de Tegucigalpa; 2002 Sep. 2-6; Tegucigalpa, MDC.

124. Gómez O, Pavón A, Mendoza S, Murillo L. Parásitos intestinales en pacientes con SIDA; 1990 Nov. 19-23; Tegucigalpa, MDC.

125. Kaminsky RG. Parasitismo intestinal en Honduras; 1991 Oct. 28 – Nov. 2; Tegucigalpa, MDC.

126. Osorio E, Canales M, Sanchez AL, Gómez O, Espinoza V, et al. Prevalencia de parásitos intestinales en niños de la Guardería Infantil "Flor del Campo"; 1987 Oct. 26-30; Tegucigalpa, MDC.

127. Quan D, Canales M. Prevalencia de geohelmintos en escolares de diferentes regiones de Honduras; 2001 Sep. 24-28; Tegucigalpa, MDC.

128. Quan D, Canales M, Espinoza V. Evaluación de la prevalencia de geohelmintos en escolares de diferentes regiones de Honduras desde 2000 al 2004; 2006 Nov. 6-10; Tegucigalpa, MDC.

129. Samayoa ME, Berlioz L, Varela C. Estudio del parasitismo intestinal en el paciente desnutrido en el Hospital Escuela; 1985 Nov. 4-8; Tegucigalpa, MDC.

130. Sanchez AL, Osorio E, Gómez O, Espinoza E, Canales M. Parásitos intestinales en escolares de una comunidad Hondureña; 1987 Oct. 26-30; Tegucigalpa, MDC.

131. Zavala A, Ruiz J. Ascaridiasis biliar. Una causa poco reconocida de enfermedad biliar y pancreática; 1989 Oct. 16-20; Tegucigalpa, MDC.

B.4 Ministry of Health national surveys

132. Honduras Ministry of Health (2003) [Survey report of soil-transmitted helminths and taeniasis in school children. 2000-2001]. Tegucigalpa, MDC. 41 p. (Report in Spanish).

133. Honduras Ministry of Health (2006) [Survey report of soil-transmitted helminths in school children in Honduras. 2005-2006]. Tegucigalpa, MDC. (Report in Spanish).

134. Honduras Ministry of Health (2011) [Prevalence of soil-transmitted helminthiases and malaria in school children. Characterization of socio-cultural and environmental risk factors. 2011]. Tegucigalpa, MDC. 179 p. (Report in Spanish).

B.5 Other governmental technical reports

135. Honduras Ministry of Natural Resources and Environment (2004) [Update on the vulnerability of the Aguán River Watershed (Locomapa) in Honduras]. Tegucigalpa, MDC. Secretaría de Recursos Naturales y Ambiente (SERNA). 179 p. (Report in Spanish).

B.6 Microbiology social service reports

136. Agüero RJ, Barahona E (1991) Informe de servicio social. Laboratorio del Hospital "Gabriela Alvarado". Danlí, El Paraiso. Marzo 1990 - Febrero 1991. Tegucigalpa, MDC.: UNAH.

137. Aguilera M (1991) Informe de servicio social. Laboratorio del Hospital Nacional Psiquiátrico "Santa Rosita". Támara, Fco. Morazán. Marzo 1990 - Febrero 1991. Tegucigalpa, MDC.: UNAH.

138. Alvarado SC (1991) Informe de servicio social. Laboratorio del Centro de Salud "Vicente Fernández Mejía". Santa Rosa de Copán, Copán. Marzo 1990 - Febrero 1991. Tegucigalpa, MDC.: UNAH.

139. Barahona CM (1990) Informe de servicio social. Laboratorio del Hospital "Dr. Roberto Suazo Córdova". La Paz, La Paz. Marzo 1989 - Febrero 1990. Tegucigalpa, MDC.: UNAH.

140. Caballero AA (2009) Informe de servicio social. Laboratorio del Centro de Salud. Nacaome, Valle. Marzo 2008 - Febrero 2009. Tegucigalpa, MDC.: UNAH.

141. Canales ML (1991) Informe de servicio social. Laboratorio del Hospital "Dr. Roberto Suazo Córdova". La Paz, La Paz. Marzo 1990 - Febrero 1991. Tegucigalpa, MDC.: UNAH.

142. Castro DN (1990) Informe de servicio social. Laboratorio del Hospital de Puerto Lempira, Gracias a Dios. Marzo 1989 - Febrero 1990. Tegucigalpa, MDC.: UNAH.

143. Enamorado NI (1992) Informe de servicio social. Laboratorio del Hospital de Área. Puerto Cortés, Cortés. Marzo 1991 - Febrero 1992. Tegucigalpa, MDC.: UNAH.

144. Escobar IG (1990) Informe de servicio social. Laboratorio del Centro de Salud. San Lorenzo, Valle. Marzo 1989 - Febrero 1990. Tegucigalpa, MDC.: UNAH.

145. Euceda S (1991) Informe de servicio social. Laboratorio del Hospital "San Francisco". Juticalpa, Olancho. Marzo 1990 - Febrero1991. Tegucigalpa, MDC.: UNAH.

146. Flores ML (1992) Informe de servicio social. Laboratorio del Hospital "Gabriela Alvarado". Danlí, El Paraíso. Marzo 1991 - Febrero 1992. Tegucigalpa, MDC.: UNAH.

147. Funes AC (1992) Informe de servicio social. Laboratorio del Hospital Psiquiátrico "Dr. Mario Mendoza". Tegucigalpa MDC, Fco. Morazán. Marzo 1991 - Febrero 1992. Tegucigalpa, MDC.: UNAH.

148. Gabrie JA (1991) Informe de servicio social. Laboratorio del Hospital "Dr. Salvador Paredes". Trujillo, Colón. Marzo1990 - Febrero 1991. Tegucigalpa, MDC.: UNAH.

149. García MO (1991) Informe de servicio social. Laboratorio del Hospital Regional "Atlántida Integrado". La Ceiba, Atlántida. Marzo 1990 - Febrero 1991. Tegucigalpa, MDC.: UNAH.

150. García NJ (1991) Informe de servicio social. Laboratorio del Centro de Salud "Dr. Gustavo Boquín". Siguatepeque, Comayagua. Marzo 1990 - Febrero 1991. Tegucigalpa, MDC.: UNAH.

151. Gutiérrez DR (1991) Informe de servicio social. Laboratorio del Centro de Salud "Dr. Constantino Silva". Nacaome, Valle. Marzo 1990 - Febrero 1991. Tegucigalpa, MDC.: UNAH.

152. Hernández CM (1991) Informe de servicio social. Laboratorio del Centro de Salud de Olanchito, Yoro. Marzo 1990 - Febrero1991. Tegucigalpa, MDC.: UNAH.

153. Laínez ME (1990) Informe de servicio social. Laboratorio del Centro de Salud "Dr. Gustavo Boquín". Siguatepeque, Comayagua. Marzo 1989 - Febrero 1990. Tegucigalpa, MDC.: UNAH.

154. Lambur SA (1990) Informe de servicio social. Laboratorio del Hospital Regional de Occidente. Santa Rosa de Copán, Copán. Marzo 1989 - Febrero 1990. Tegucigalpa, MDC.: UNAH.

155. Lara SM (1992) Informe de servicio social. Laboratorio del Hospital Nacional Psiquiátrico "Santa Rosita". Támara, Fco. Morazán. Marzo 1991 - Febrero 1992. Tegucigalpa, MDC.: UNAH.

156. Leán M (1992) Informe de servicio social. Laboratorio del Centro de Salud "Dr. Miguel Paz Barahona". San Pedro Sula, Cortés. Marzo 1991 - Febrero 1992. Tegucigalpa, MDC.: UNAH.

157. Martínez NG (1990) Informe de servicio social. Laboratorio del Hospital de Área "Gabriela Alvarado". Danlí, El Paraíso. Marzo 1989 - Febrero 1990. Tegucigalpa, MDC.: UNAH.

158. Mass E (1991) Informe de servicio social. Laboratorio del Hospital de Área. Puerto Cortés, Cortés. Marzo 1990 - Febrero 1991. Tegucigalpa, MDC.: UNAH.

159. Mejía ZE (1990) Informe de servicio social. Laboratorio del Centro de Salud "Dr. Constantino Silva". Nacaome, Valle. Marzo 1989 - Febrero 1990. Tegucigalpa, MDC.: UNAH.

160. Molina YZ (1992) Informe de servicio social. Laboratorio del Hospital "San Francisco". Juticalpa, Olancho. Marzo 1991 - Febrero 1992. Tegucigalpa, MDC.: UNAH.

161. Morán ER (1990) Informe de servicio social. Laboratorio del Hospital "Dr. Salvador Paredes". Trujillo, Colón. Marzo 1989 - Febrero 1990. Tegucigalpa, MDC.: UNAH.

162. Murcia M (1990) Informe de servicio social. Laboratorio del Centro de Salud "Dr. Luis Lazo Arriaga". El Paraiso, El Paraiso. Marzo 1989 - Febrero 1990. Tegucigalpa, MDC.: UNAH.

163. Núñez A (1992) Informe de servicio social. Laboratorio del Hospital "Dr. Salvador Paredes". Trujillo, Colón. Marzo 1991 - Febrero 1992. Tegucigalpa, MDC.: UNAH.

164. Núñez KM (1991) Informe de servicio social. Laboratorio del Hospital de Puerto Lempira, Gracias a Dios. Marzo 1990 - Febrero 1991. Tegucigalpa, MDC.: UNAH.

165. Pineda LA (1991) Informe de servicio social. Laboratorio del Hospital "Tela Integrado". Tela, Atlántida. Marzo 1990 - Febrero 1991. Tegucigalpa, MDC.: UNAH.

166. Pineda LJ (1991) Informe de servicio social. Laboratorio del Hospital "Dr. Leonardo Martínez", Marzo 1990 - Octubre 1990 y del Hospital "Dr. Mario Catarino Rivas", Octubre 1990 - Febrero 1991. San Pedro Sula, Cortés. Tegucigalpa, MDC.: UNAH.

167. Reithel CR (1991) Informe de servicio social. Laboratorio Región Sanitaria No. 2. Comayagua, Comayagua. Marzo 1986 - Febrero 1987. Tegucigalpa, MDC.: UNAH.

168. Rodríguez AL (1991) Informe de servicio social. Laboratorio de la Región Sanitaria No. 7. Juticalpa, Olancho. Marzo 1990 - Febrero1991. Tegucigalpa, MDC.: UNAH.

169. Romero EA (1992) Informe de servicio social. Laboratorio del Hospital Regional de Occidente. Santa Rosa de Copán, Copán. Marzo 1991 - Febrero 1992. Tegucigalpa, MDC.: UNAH.

170. Romero IJ (1991) Informe de servicio social. Laboratorio del Hospital de Área "El Progreso". El Progreso, Yoro. Marzo 1990 - Febrero1991. Tegucigalpa, MDC.: UNAH.

171. Soto F (1990) Informe de servicio social. Laboratorio del Hospital Nacional Psiquiátrico "Santa Rosita". Támara, Fco. Morazán. Marzo 1989 - Febrero 1990. Tegucigalpa, MDC.: UNAH.

172. Urbina AI (1991) Informe de servicio social. Laboratorio del Hospital "Manuel de Jesús Subirana". Yoro, Yoro. Marzo 1990 - Febrero 1991. Tegucigalpa, MDC.: UNAH.

173. Wong LM (1990) Informe de servicio social. Laboratorio del Centro de Salud "Dr. Gregorio A. Lobo". Catacamas, Olancho. Marzo 1989 - Febrero 1990. Tegucigalpa, MDC.: UNAH.

B.7 UNAH’s Parasitology course field trips

174. Canales M (1999) Prevalencia de geohelmintos en niños del albergue "El Trebol". Junio 1999.

175. Canales M (2003) Prevalencia de geohelmintos en las comunidades de Nueva Armenia, Corozal y El Porvenir. Diciembre 2003.

176. Canales M (2004) Prevalencia de geohelmintos en niños de la comunidad de La Bolsita, Sta. Cruz de Yojoa. Octubre 2004.

177. Canales M (2004) Prevalencia de geohelmintos en niños de la comunidad de El Ocotal, Sta. Cruz de Yojoa. Octubre 2004.

178. Canales M (2004) Prevalencia de geohelmintos en niños de la comunidad de Cónica, Sta. Cruz de Yojoa. Octubre 2004.

179. Canales M (2004) Prevalencia de geohelmintos en la comunidad de Terreritos, Comayagua. Noviembre 2004.

180. Canales M (2004) Prevalencia de geohelmintos en la comunidad de La Bolsita, Sta. Cruz de Yojoa. Noviembre 2004.

181. Canales M (2006) Prevalencia de geohelmintos en niños de la comunidad de Dulce Nombre de Copán. Mayo 2006.

182. Canales M (2006) Prevalencia de geohelmintos en niños de la comunidad de Casa Blanca, La Ceiba. Noviembre 2006.

183. Canales M (2007) Prevalencia de geohelmintos en la escuela de niñas José Cecilio del Valle, Olanchito. Junio 2007.

184. Canales M (2007) Prevalencia de geohelmintos en niños de la comunidad de Olanchito. Junio 2007.

185. Canales M (2007) Prevalencia de geohelmintos en la comunidad de El Chile, Puerto Cortés. Agosto 2007.

186. Canales M (2007) Prevalencia de geohelmintos en niños de la escuela Puertas del Renacimiento, Aldea Culuco, Gualaco. Octubre 2007.

187. Canales M (2007) Prevalencia de geohelmintos en la comunidad de Coronado, San Esteban. Diciembre 2007.

188. Canales M (2008) Prevalencia de geohelmintos en niños de la comunidad de Linaca, Tatumbla. Mayo 2008.

189. Canales M (2008) Prevalencia de geohelmintos en niños de la comunidad de El Picacho. Octubre 2008.

190. Canales M (2009) Prevalencia de geohelmintos en niños de la escuela rural mixta "Tim Hands", Villa Vieja, Col. Mirador de Oriente. Octubre 2009.

191. Canales M (2009) Prevalencia de geohelmintos en niños de la escuela rural mixta "Tim Hands", Villa Vieja, Colonia Mirador de Oriente. Diciembre 2009.

192. Canales M (2010) Prevalencia de geohelmintos en niños del centro de educación básica "La Fraternidad", Comayagüela MDC. Mayo 2010.

193. Canales M (2010) Prevalencia de geohelmintos en niños de la escuela "Lisandro Quezada", Tegucigalpa MDC. Mayo 2010.

194. Canales M (2010) Prevalencia de geohelmintos en niños del centro de educación básica "Ramiro H. Moreno", Comayagua. Mayo 2010.

195. Canales M (2010) Prevalencia de geohelmintos en niños de la escuela "Pedro Nufio", Aldea Cerro Grande. Mayo 2010.

196. Canales M (2010) Prevalencia de geohelmintos en la comunidad Pech, Subirana. Octubre 2010.

197. Canales M (2011) Prevalencia de geohelmintos en niños del centro de educación básica "Luis Alonso Cruz Escalante", Aldea Sta. Cruz, Comayagüela MDC. Mayo 2011.

198. Canales M (2011) Prevalencia de geohelmintos en niños del centro de educación básica "Manuel Bonilla", Aldea Cerro Grande, Valle de Ángeles. Mayo 2011.

199. Canales M (2011) Prevalencia de geohelmintos en niños de la escuela "José Cecilio del Valle", Los Laureles, Sta. Bárbara. Octubre 2011.

200. Canales M (2011) Prevalencia de geohelmintos en niños de la escuela "Leovigildo Pineda", La Fe, Sta. Bárbara. Noviembre 2011.

201. Canales M, Quan D, Espinoza V (2004) Prevalencia de geohelmintos en niños de comunidades de la cuenca del río Aguán, Guata, Olancho. Mayo 2004.

202. Hernández K, Canales M (2009) Prevalencia de geohelmintos en niños del centro básico Esteban Guardiola, El Triunfo de la Cruz. Mayo 2009.

203. Jovel I, Canales M (2004) Prevalencia de geohelmintos en la comunidad de Sta. Cruz de Yojoa. Octubre 2004.

204. Jovel I, Quan D, Espinoza E (2008) Prevalencia de geohelmintos en niños de la escuela Cub de Leones, Las Crucitas. Agosto 2008.

205. Jovel I, Quan D, Espinoza E (2009) Prevalencia de geohelmintos en niños del centro de educación básica "Manuel Bonilla", Aldea Cerro Grande, Valle de Ángeles. Agosto 2009.

206. Quan D (2011) Prevalencia de geohelmintos en niños del centro educativo experimental UNAH, Tegucigalpa MDC. Septiembre 2011.

207. Quan D (2012) Prevalencia de geohelmintos en niños de la escuela "Nora Gúnera de Melgar", Tegucigalpa MDC. Abril 2012.

208. Quan D, Canales M, Espinoza E (2012) Prevalencia de geohelmintos en las comunidades de La Barquera y La Isla, San José de Colinas, Sta. Bárbara. Abril 2012.

209. Quan D, Rodríguez C (2011) Prevalencia de geohelmintos en niños de la escuela "Gonzalo Mauricio Alvarado", San Lorenzo, Valle. Noviembre 2011.

210. Sosa W (2010) Prevalencia de geohelmintos en niños del centro de educación básica "Altagracia Sánchez Méndez", Cataluña, Tegucigalpa MDC. Agosto 2010.

211. Sosa W (2010) Prevalencia de geohelmintos en niños del centro de educación básica "La Joya", Támara. Agosto 2010.
